# Structure-based design of a disulfide-linked oligomeric form of the simian virus 40 (SV40) large T antigen DNA-binding domain

**DOI:** 10.1107/S0907444911014302

**Published:** 2011-05-17

**Authors:** Gretchen Meinke, Paul Phelan, Amélie Fradet-Turcotte, Jacques Archambault, Peter A. Bullock

**Affiliations:** a Department of Biochemistry, Tufts School of Medicine and the Sackler School of Graduate Biomedical Sciences, 136 Harrison Avenue, Boston, MA 02111, USA; b Laboratory of Molecular Virology, Institut de Recherches Cliniques de Montréal (IRCM), Montreal, Quebec, Canada

**Keywords:** SV40 large T antigen, origin-binding domain, disulfides, oligomerization, DNA replication

## Abstract

The modular multifunctional protein large T antigen (T-ag) from simian virus 40 orchestrates many of the events needed for replication of the viral double-stranded DNA genome. This protein assembles into single and double hexamers on specific DNA sequences located at the origin of replication. This complicated process begins when the origin-binding domain of large T antigen (T-ag ODB) binds the GAGGC sequences in the central region (site II) of the viral origin of replication. While many of the functions of purified T-ag OBD can be studied in isolation, it is primarily monomeric in solution and cannot assemble into hexamers. To overcome this limitation, the possibility of engineering intermolecular disulfide bonds in the origin-binding domain which could oligomerize in solution was investigated. A recent crystal structure of the wild-type T-ag OBD showed that this domain forms a left-handed spiral in the crystal with six subunits per turn. Therefore, we analyzed the protein interface of this structure and identified two residues that could potentially support an intermolecular disulfide bond if changed to cysteines. SDS–PAGE analysis established that the mutant T-ag OBD formed higher oligomeric products in a redox-dependent manner. In addition, the 1.7 Å resolution crystal structure of the engineered disulfide-linked T-ag OBD is reported, which establishes that oligomerization took place in the expected manner.

## Abstract 2

With the aim of forming the ‘lock-washer’ conformation of the origin-binding domain of SV40 large T antigen in solution, using structure-based analysis an intermolecular disulfide bridge was engineered into the origin-binding domain to generate higher order oligomers in solution. The 1.7 Å resolution structure shows that the mutant forms a spiral in the crystal and has the *de novo* disulfide bond at the protein interface, although structural rearrangements at the interface are observed relative to the wild type.

## 1. Introduction

Simian virus 40 (SV40) is a member of the polyoma virus family and has long served as a model system for the study of eukaryotic DNA replication (for reviews, see Bullock, 1997 ▶; Fanning & Knippers, 1992 ▶; Simmons, 2000 ▶). In this virus, replication depends upon a single viral protein, large T antigen (T-ag), with the other necessary factors being provided by the cellular host.

The site at which DNA replication initiates, termed the origin of replication, contains three important regions: a central region with four pentameric GAGGC sequences arranged as two pairs of inverted repeats (termed site II) and two flanking regions termed the AT-rich region and the EP region (Deb *et al.*, 1986 ▶). For SV40, this minimal 64-base-pair origin sequence, termed the core origin, is sufficient to support T-ag assembly and initiation of viral DNA replication (reviewed in Borowiec *et al.*, 1990 ▶). Electron-microscopy studies have shown that upon binding the core origin T-ag molecules assemble into head-to-head double hexamers (Mastrangelo *et al.*, 1989 ▶; Valle *et al.*, 2000 ▶).

T-ag contains three structurally and functionally independent domains: an N-terminal J domain followed by the origin-binding domain (OBD) and the helicase domain. The J domain, while necessary for transformation (reviewed in Pipas, 2009 ▶), is not essential for replication *in vitro* (Campbell *et al.*, 1997 ▶). The T-ag OBD has multiple functions (Wun-Kim *et al.*, 1993 ▶); it binds the GAGGC sequences in the origin of replication in a sequence-specific manner. The T-ag OBD can also bind nonspecific dsDNA and ssDNA (see, for example, Fradet-Turcotte *et al.*, 2007 ▶; Reese *et al.*, 2006 ▶; Simmons, Loeber *et al.*, 1990 ▶; Simmons, Wun-Kim *et al.*, 1990 ▶).

The helicase domain belongs to helicase superfamily 3 (SF3) and is a member of the AAA+ (ATPases associated with diverse cellular activities) family (for a review, see Erzberger & Berger, 2006 ▶). The T-ag helicase forms hexameric ring structures that work as molecular motors to unwind DNA using ATP hydrolysis (Li *et al.*, 2003 ▶).

Crystal and NMR structures are available for each of the individual domains in a variety of states, although no high-resolution structure yet exists for the full-length protein. For example, crystal structures of the T-ag OBD on DNA con­taining the four GAGGC sequences (Bochkareva *et al.*, 2006 ▶), as well as on a pair of GAGGC sequences (Meinke *et al.*, 2007 ▶), have been solved. Crystal structures also exist of T-­ag OBD interacting with dsDNA in a nonsequence-specific manner (Bochkareva *et al.*, 2006 ▶; Meinke *et al.*, 2011 ▶) and a recent crystal structure shows how T-ag OBD interacts with the ssDNA portion of a forked-DNA substrate (Meinke *et al.*, 2011 ▶).

Owing to the modular nature of T-ag, many of its functions may be studied by examining the isolated domains. For example, the helicase domain has ATPase activity similar to that of the full-length protein (Gai *et al.*, 2004 ▶). In the case of the T-ag OBD, many of its DNA-binding properties have been measured using the purified isolated domain (see, for example, Fradet-Turcotte *et al.*, 2007 ▶; Joo *et al.*, 1997 ▶; Reese *et al.*, 2006 ▶; Titolo *et al.*, 2003 ▶). However, unlike the helicase domain, which oligomerizes readily into hexamers in the presence of ADP, ATP or an analog, the isolated OBD remains monomeric in solution even at concentrations of >1 m*M* (Luo *et al.*, 1996 ▶).

Our structure of the T-ag OBD (PDB entry 2fuf) crystallized in a hexagonal space group in which the protein formed a helical filament in the crystal with six monomers per turn (Meinke *et al.*, 2006 ▶). We proposed that a single turn of the helical filament (which we term the open-ring or ‘lock-washer’ structure) may be biologically relevant. Furthermore, we proposed that it would form after recognition of the GAGGC sequences by the T-ag OBD (Meinke *et al.*, 2007 ▶) and subsequent assembly events by the helicase domain (Kumar *et al.*, 2007 ▶). This ‘open-ring’ structure of the T-ag OBD has a positively charged central channel that is large enough to accommodate dsDNA or even two opposing strands of ssDNA. The ‘gap’ in the open ring may also provide a path through which ssDNA can pass. Furthermore, this structure provided the first high-resolution view of the residues in T-­ag OBD that are involved in the protein–protein interactions required for hexamer formation. Of note, the overall dimensions of the spiral hexamer T-ag OBD structure fit those seen in cryo-EM studies of larger constructs of T-ag assembled on origin-containing DNA substrates (Cuesta *et al.*, 2010 ▶; Valle *et al.*, 2000 ▶, 2006 ▶).

Because the full-length T-ag functions as a hexamer, we wished to engineer a ring form of the isolated T-ag OBD. Using structure-based design, we identified a pair of residues at the protein–protein interface that were predicted to support disulfide-bond formation upon mutation to cysteines. We generated this mutant form of T-ag OBD and established that it formed the predicted higher order complexes in a redox-dependent manner. To validate the hypothesis that an intermolecular disulfide bond formed at the mutated positions within the spiral structure, we solved the crystal structure of this mutant T-ag OBD containing a pair of engineered cysteines. These data support the hypothesis that the disulfide-linked T-ag OBDs form a spiral in solution.

## 2. Materials and methods

### 2.1. Structure-based design of a disulfide bond

The program *SSBOND* (Hazes & Dijkstra, 1988 ▶) analyzes protein structures and identifies pairs of residues that can support disulfide-bond formation if mutated to cysteines. We used *SSBOND* to analyze the protein–protein interface of the SV40 T-ag OBD open-ring hexamer crystal structure (PDB entry 2fuf). The program identified only one possible pair of residues (Phe151 and Asp256) as having the potential to form a disulfide bond. In addition, analysis of the wild-type (wt) T-­ag OBD structure revealed that only one of the three existing cysteines in the wt T-ag OBD structure is solvent-accessible (Cys216) and thus a potential problem for these analyses.

### 2.2. Vector construction

#### 2.2.1. Generation of mutant T-ag OBD

The pGEX TBD vector expresses wild-type SV40 T-ag OBD_131–260_ (amino acids 131–260) as a thrombin-cleavable GST-fusion protein and has been described elsewhere (Joo *et al.*, 1997 ▶). Alanine or cysteine substitutions were introduced at targeted positions using the QuikChange site-directed mutagenesis kit (Stratagene), plasmid pGEX TBD and appropriate DNA oligo­nucleotides (shown in Table 1 ▶). The thermocycling conditions consisted of an initial denaturation step at 368 K for 60 s followed by 12 cycles of denaturation at 368 K for 50 s, annealing at 328 K for 60 s, polymerase extension at 341 K for 11 min and a final extension of 5 min. The DNA sequences of all mutant plasmids described in this report were confirmed by the dideoxy sequencing method at the Tufts Core Facility. The following four T-ag OBD mutants were produced: (i) T-ag OBD_C216A_, (ii) T-ag OBD_C216A, F151C_, (iii) T-ag OBD_C216A, D256C_ and (iv) T-ag OBD_C216A, F151C, D256C_.

#### 2.2.2. Generation of mutant full-length T-ag

Plasmid pCMVneoT-ag (Campbell *et al.*, 1997 ▶) was used for expression of T-ag. Mutant forms of full-length T-ag were prepared using the QuikChange kit (Stratagene) with pCMVneoT-ag as the sub­strate and the primers listed in Table 1 ▶. The thermocycling conditions were identical to those listed above.

### 2.3. Protein expression and purification

The T-ag OBD mutants were expressed and purified in a similar manner as the wild type (Meinke *et al.*, 2006 ▶). Briefly, upon induction with IPTG (isopropyl β-d-1-thiogalacto­pyranoside), the GST fusion was overexpressed in *Escherichia coli* strain BL21 (DE3). The cells were lysed and spun down and the GST fusion was purified on a glutathione Sepharose 4B column. This was followed by digestion with thrombin and two further chromatographic steps: cation exchange (Source S resin) followed by gel filtration. The T-ag OBD mutants were purified as described previously, with the exception that the Sephacryl S-100 column was replaced by a Superdex 75 10/300 GL column. Upon purification to apparent homogeneity, the T-ag OBD mutants were stored in storage buffer (20 m*M* Tris–HCl pH 8.0, 50 m*M* NaCl, 0.1% β-mercaptoethanol and 10% glycerol) at 193 K until ready for use.

### 2.4. Cysteine–cysteine cross-linking catalyzed by Cu(1,10-phenanthroline)_2_SO_4_ (CuP)

The phenanthroline copper complex has been shown to catalytically oxidize sulfhydryl groups (Kobashi, 1968 ▶). The cross-linking protocol described here is adapted from Jiang & Fillingame (1998 ▶). The stock protein (wt or mutant T-ag OBD) was diluted to 1 mg ml^−1^ in 1× reaction buffer (10 m*M* Tris pH 7.5, 100 m*M* NaCl, 5% glycerol). A fresh 10× Cu(1,10-phenanthroline)_2_SO_4_ (CuP) solution was prepared by making a 15 m*M* CuSO_4_, 50 m*M* 1,10-phenanthroline solution.

Each 50 µl cross-linking reaction contained 10 µg protein (unless otherwise indicated) in 1× reaction buffer to which a1/10 volume of 10× CuP was added. This mixture was allowed to incubate for 60 min at room temperature.

The cross-linking reaction was terminated by the addition of EDTA to a final concentration of 50 m*M* and the addition of *N*-­ethylmaleimide to a final concentration of 2 m*M*. After 15 min at room temperature, a 1/6 volume of 6× SDS sample buffer (350 m*M* Tris–HCl pH 6.8, 10% SDS, 30% glycerol, 0.12 mg ml^−1^ bromophenol) was added to the reaction. This mixture was allowed to incubate at room temperature for 1 h. If the cross-link was to be reduced, DTT was then added to a final concentration of 25 m*M*. The products were then electro­phoresed on 12–15% SDS gels and analyzed *via* Coomassie staining.

### 2.5. Luciferase transient DNA-replication assay

A quantitative assay to study the replication of polyoma and papilloma viruses has recently been described (Fradet-Turcotte *et al.*, 2010 ▶). This assay measures the ability of full-length or mutants of T-ag to replicate an origin-containing plasmid that also has a luciferase reporter gene. We generated T-ag containing mutations at residues in the spiral interface of the T-ag OBD involved in disulfide-bond formation and subjected them to this assay. The following three mutants were made in the parental pCMVneoT-ag expression vector: (i) F151C, (ii) D256C and (iii) F151C, D256C.

To perform the assay, 5 × 10^4^ C33A cells were transfected with three plasmids encoding T-ag, the minimal origin of DNA replication together with a firefly luciferase reporter gene and *Renilla* luciferase. Replication of the origin-containing plasmid was quantified 72 h post-transfection by measuring the levels of firefly and *Renilla* luciferase activities using a Dual-Glo Luciferase assay system (Promega). Each T-­ag mutant was analyzed in duplicate in two separate experiments. Error bars represent the standard deviations.

### 2.6. Western blotting assay

Western blotting was performed *via* standard methods; extracts were prepared 24 h post-transfection. T-ag proteins were detected using a mouse monoclonal antibody recognizing an epitope located within the N-terminal 82 amino acids of the protein (Sc-148; Santa Cruz). Tubulin was used as a loading control.

### 2.7. Crystallization of the SV40 T-ag OBD triple mutant (F151C, C216A, D256C)

Crystallization of the T-ag OBD_F151C, C216A, D256C_ triple mutant (3XM) was performed by screening around conditions that previously yielded diffraction-quality crystals. Initial microcrystals were grown under conditions similar to the published conditions for the wt T-ag OBD hexagonal crystal form (Meinke *et al.*, 2006 ▶), although growing single crystals of the triple mutant proved difficult. It was previously observed that wt T-ag OBD hexagonal crystals were also obtained in the presence of excess single-stranded DNA (ssDNA; unpublished data). Therefore, ssDNA (12-mer of poly-dT resuspended in 10 m*M* Tris pH 7.5, 50 m*M* NaCl; Integrated DNA Technologies) was added in a molar ratio of 2:1 to the 3XM protein to help grow larger crystals. In general, crystals of the triple mutant grew as clusters of small crystals which had to be cut using a microtool to obtain a single crystal. The best crystals were obtained by the vapour-diffusion method at room temperature in a 24-well Linbro tray. The 1 ml well solution consisted of 1.6 *M* sodium citrate pH 6.5. The crystals were grown from drops made up of 3 µl protein solution (6.4 mg ml^−1^ 3XM and a twofold molar excess of 12-mer poly-dT in protein storage buffer) and 4 µl well solution.

### 2.8. X-ray data collection, structure solution and refinement

The crystals were transferred to a cryogenic solution using a nylon microloop (Hampton Research) and then frozen in liquid nitrogen. The cryogenic solution used for the T-ag OBD triple mutant consisted of 1.36 *M* sodium citrate pH 6.5 and 15% ethylene glycol. X-ray data were collected at 100 K using an ADSC Quantum-315r nine-quadrant charge-coupled device (CCD) detector on National Synchrotron Light Source (NSLS) beamline X-29 (Brookhaven National Laboratory, New York, USA). The X-ray data were processed with the *HKL*-2000 suite (Otwinowski & Minor, 1997 ▶). The unit-cell parameters and diffraction statistics are shown in Table 2 ▶. The structure of the triple mutant was solved by molecular replace­ment using apo T-ag OBD coordinates as a search model (PDB entry 2fuf) and the program *Phaser* (McCoy *et al.*, 2005 ▶). The structure was refined using *REFMAC* (Murshudov *et al.*, 2011 ▶) within the *CCP*4 suite of programs (Winn *et al.*, 2011 ▶) and *PHENIX* (Adams *et al.*, 2010 ▶). Four TLS (translation/libration/screw) groups were identified (Painter & Merritt, 2006 ▶) and were included in the final steps of refinement. The resulting maps and models were visualized and improved with the molecular-graphics programs *Coot* (Emsley *et al.*, 2010 ▶) and *PyMOL* (Schrodinger LLC). *PDBSUM* was used to analyze the protein–protein interface (Laskowski, 2009 ▶). All molecular-graphics figures in this paper were produced with *PyMOL*.

## 3. Results and discussion

### 3.1. Structural analysis of the spiral hexamer of wt SV40 T-ag OBD

As previously mentioned, the T-ag OBDs interact with each other when the full-length T-ag forms single or double hexamers, whereas in solution the purified isolated OBD is primarily monomeric.

**Figure 1:**
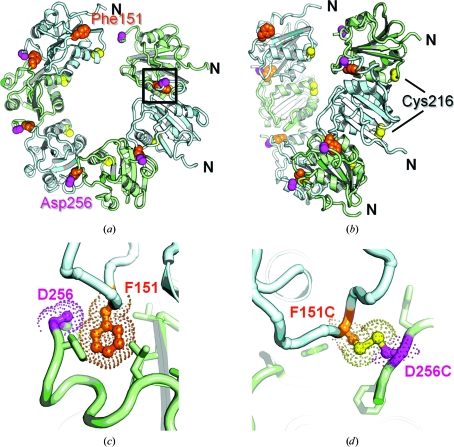
Crystal structure of the spiral hexamer structure of wt SV40 T-ag OBD shown as a ribbon diagram. (*a*) This ‘top’ view is looking down the sixfold axis. A close-up view of the area bounded by the black box is shown in (*c*). (*b*) This ‘side’ view is rotated ∼90° relative to the view in (*a*) and clearly shows the ‘gap’. Alternating monomers of T-ag OBD are colored cyan and green, respectively. The residues Phe151, Cys216 and Asp256 are shown as orange, yellow and magenta spheres, respectively. (*c*) A close-up view of the interface showing Phe151 (orange) and Asp256 (magenta) as sticks. Note that there are no side chains modelled for the Asp256 residue. (*d*) A molecular model of the disulfide bridge that could form between F151C and D256C. The S atoms are colored yellow.

We have shown that wt T-ag OBD forms a filament in the crystal (Meinke *et al.*, 2006 ▶). This crystal structure of wt SV40 T-ag OBD (PDB entry 2fuf) has one monomer in the asymmetric unit and six monomers per turn (Figs. 1 ▶ *a* and 1 ▶ *b*). The monomers pack in a head-to-­tail arrangement. We analyzed the protein–protein interface of this high-resolution crystallographic hexamer using *SSBOND* (Hazes & Dijkstra, 1988 ▶). *SSBOND* predicts which pairs of residues, if mutated to cysteines, could reasonably form disulfide bonds. When the wt T-ag OBD structure was analyzed *via* the program *SSBOND*, we identified only one pair as candidates for mutation: Phe151 and Asp256. The positions of these residues are shown in Figs. 1 ▶(*a*) and 1 ▶(*b*) and a close-up view is shown in Fig. 1 ▶(*c*). A theoretical model of the disulfide linkage is shown in Fig. 1 ▶(*d*). Further visual inspection of the structure of T-ag OBD revealed one surface cysteine (Cys216; shown in Figs. 1 ▶ *a* and 1 ▶ *b*). Indeed, the crystal structure of a Cys216–Cys216 disulfide-linked T-ag OBD dimer has been reported (PDB entry 2if9; Meinke *et al.*, 2007 ▶). Therefore, to eliminate disulfide formation with Cys216, we also mutated Cys216 to alanine (Cys216Ala).

### 3.2. Cys216Ala T-ag OBD is monomeric

**Figure 2:**
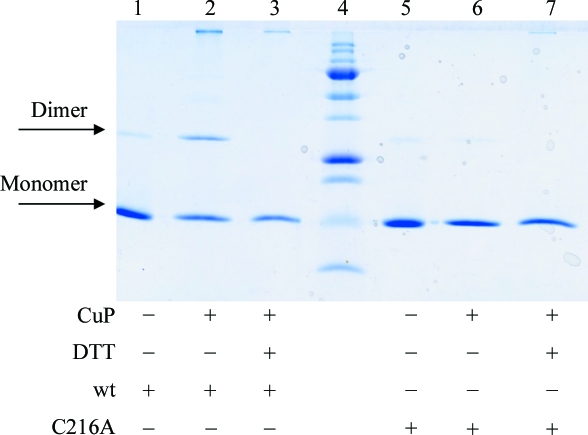
T-ag OBD Cys216A mutant is monomeric in solution. Results of Cu(1,10-phenanthroline)_2_SO_4_ (CuP) catalyzed oxidation experiments electrophoresed on an SDS–polyacrylamide gel and stained with Coomassie Brilliant Blue. Lane 4 contains molecular-weight markers (250, 150, 100, 75, 50, 37, 25, 20, 15 and 10 kDa). Lanes 1–3 contain wt T-ag OBD and lanes 5–7 contain the C216A mutant protein. Lanes 2 and 6 show the results of CuP oxidation and lanes 3 and 7 show the results of oxidation with CuP followed by treatment with the reducing agent DTT. These data show that under oxidizing conditions the wt OBD forms dimers, whereas the C216A mutant remains monomeric.

The wt T-ag OBD is monomeric under reducing conditions and remains as a monomer even at high concentrations (Luo *et al.*, 1996 ▶). However, as shown in Fig. 2 ▶, under oxidizing conditions some of the wt T-ag OBD forms dimers, whereas the Cys216Ala mutant does not. This confirms the hypothesis that under oxidizing conditions the surface Cys216 is responsible for the formation of disulfide-linked dimers of wt T-ag OBD.

### 3.3. Double mutants form redox-dependent dimers; the triple mutant forms redox-dependent higher order oligomers

**Figure 3:**
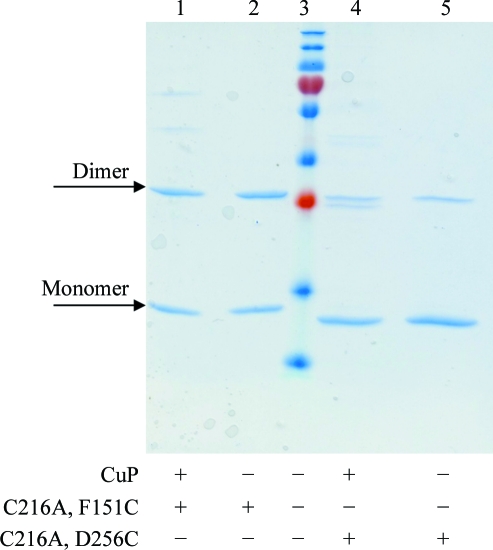
T-ag OBD double mutants primarily form dimers. Results of Cu(1,10-phenanthroline)_2_SO_4_ (CuP) catalyzed oxidation experiments electrophoresed on an SDS-polyacrylamide gel and stained with Coomassie Brilliant Blue. Lane 3 contains molecular-weight markers (250, 150, 100, 75, 50, 37, 25, 20, 15 and 10 kDa). Lanes 1–2 show C216A, F151C mutant OBD and lanes 4–5 show C216A, D256C mutant protein. Lanes 1 and 4 show the results of CuP oxidation and lanes 2 and 5 show the results of overnight dialysis in reaction buffer (no reducing agent). These data show that under oxidizing conditions the double mutants form dimers, unlike the single C216A mutant.

**Figure 4:**
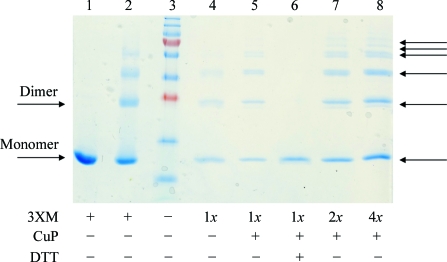
T-ag OBD triple mutants form ladders. Results of Cu(1,10-phenanthroline)_2_SO_4_ (CuP) catalyzed oxidation experiments electrophoresed on an SDS-polyacrylamide gel and stained with Coomassie Brilliant Blue. Lane 3 contains molecular-weight markers (250, 150, 100, 75, 50, 37, 25, 20, 15 and 10 kDa). Lanes 4–8 contain varying amounts of the triple mutant (3XM): 1*x*, 2*x* or 4*x*, where *x* = 1 mg ml^−1^. Lane 1 contains the reduced 3XM protein and lanes 2 and 4 show the 3XM protein after overnight dialysis in reaction buffer in the absence of reducing agent. Lane 5 shows the results of CuP oxidation. Lane 6 shows the results of oxidation followed by the addition of DTT. Lanes 7 and 8 are similar to lane 5 but with increasing concentrations of 3XM. The arrows indicate higher order species (*e.g.* monomers, dimers, trimers *etc*.). These data show that under oxidizing conditions (either by dialysis or treatment with CuP), the triple mutant forms ladders (to a much greater extent than the wild type) that can be removed by treatment with the reducing agent DTT.

Using the vector encoding the Cys216Ala mutant (pGEX T-­ag OBD_C216A_), we generated two additional vectors encoding the double mutants Cys216Ala, Phe151Cys and Cys216Ala, Asp256Cys. The mutant T-ag OBDs encoded by these vectors were purified and analyzed; these double mutants primarily formed dimers under oxidizing conditions, as shown in Fig. 3 ▶. Finally, using a vector encoding a double mutant of T-ag OBD (pGEX T-ag OBD_C216A, F151C_), we generated a vector encoding the triple-mutant form T-ag OBD_F151C, C216A, D256C_ (termed 3XM). Fig. 4 ▶ shows that the triple mutant behaves like the wild type under reducing conditions (*i.e.* it is a monomer). However, under oxidizing conditions 3XM forms higher order complexes (dimers, trimers *etc*.) as analyzed by SDS–PAGE (Fig. 4 ▶). These higher order complexes are observed by overnight dialysis in the absence of reducing agent (data not shown), as well as by using the copper-catalyzed cross-linking protocol described in §[Sec sec2]2. These higher order complexes disappear on the addition of a reducing agent such as DTT (lane 6, Fig. 4 ▶). Only the triple mutant containing both cysteines had a strong propensity to form higher order complexes.

### 3.4. Testing the ability of full-length T-ag molecules with cysteine mutations in the OBD to catalyze DNA replication

**Figure 5:**
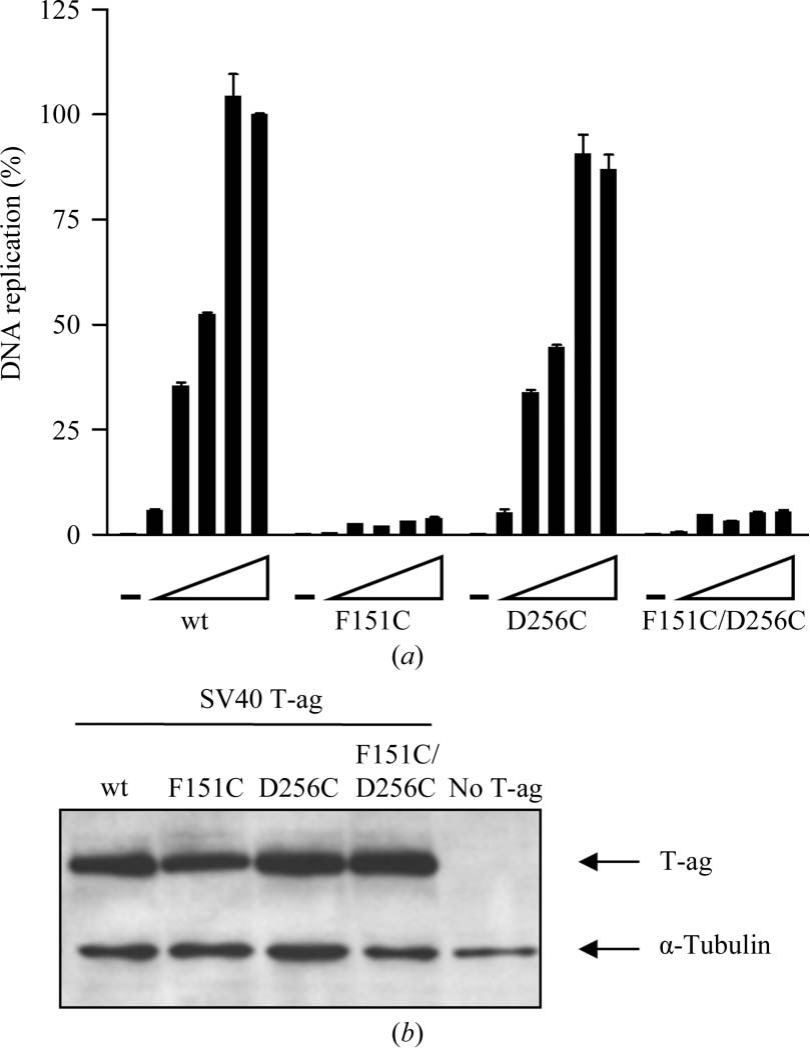
DNA-replication activity of T-ag mutant proteins. (*a*) Transient DNA-replication activities of the indicated wt or mutant T-ag proteins in C33A cells. DNA-replication activities were measured by determining the ratio of firefly (Fluc-ori plasmid) to *Renilla* (Rluc control plasmid) luciferase activities as described in §[Sec sec2]2. Replication activities are reported as a percentage of the Fluc:Rluc ratio obtained with the largest amount of wt T-ag expression vector (12 ng). Cells transfected with vector only (No T-­ag) were used as a negative control. (*b*) Expression of T-ag proteins. Western blot analysis of total protein extracts prepared from transfected C33A cells expressing the wt or the indicated T-ag mutant proteins. Each protein was tested using a gradient of expression vector (0, 1.25, 2.5, 5.0, 10 and 12.5 ng). 100% replication was defined as the amount of replication observed using 12.5 ng wt T-ag.

The cysteine mutations were introduced into vectors encoding full-length T-ag (§[Sec sec2]2) and their ability to support DNA replication was analyzed *in vivo* in transfected cells (Fig. 5 ▶). Residue Phe151 has previously been shown to be important for a variety of T-ag’s functions (see, for example, Meinke *et al.*, 2011 ▶), so it is not surprising that the F151C mutant is severely impaired for replication. In contrast, the D256C mutation has no effect on replication; this residue is located in the flexible hinge region between the OBD and the helicase domain, which may explain why this mutation is tolerated. Finally, the double mutant (F151C, D256C) was also inactive, presumably owing to the F151C mutation.

### 3.5. Crystal structure of the T-ag OBD triple mutant (3XM)

To verify that the desired intermolecular disulfide bond formed between Phe151Cys and Asp256Cys, we crystallized the triple mutant and subjected the crystals to X-ray analysis.

**Figure 6:**
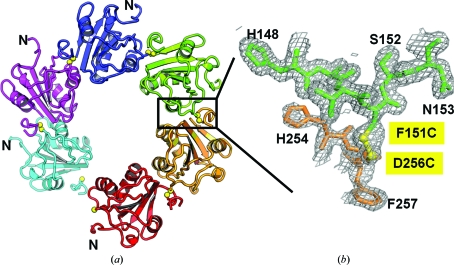
(*a*) A ribbon diagram of the T-ag OBD triple-mutant hexamer structure looking down the sixfold axis. The different protomers are colored differently. The disulfide linkage is indicated by yellow spheres. The N-­termini are labelled. A box is drawn around the region used in the close-up view. (*b*) A close-up of the 2*F*_o_ − *F*_c_ σ_A_-weighted electron-density map contoured at 0.8σ around the protein–protein interface. Again, the different protomers are colored differently and the residues are shown as sticks and labeled. The disulfide bond is shown in yellow.

The triple mutant crystallized under similar conditions to wt T-ag OBD. The crystals were much smaller and had to be dissected from a larger cluster. As previously noted, single-stranded DNA (ssDNA) was added to improve the crystals. No ordered ssDNA was visible in the electron density and there is no DNA in the model. The triple mutant crystallized in the same space group (*P*6_5_) and has almost identical unit-cell parameters as the wt T-ag OBD. There is one 3XM molecule in the asymmetric unit. Data were collected to 1.66 Å resolution and refined to a final *R* factor of 17.0% and *R*_free_ of 19.1%. The final model contains amino acids 131–252 and 254–257, 127 water molecules and one citrate molecule. Residues lacking side-chain electron density are modelled as ‘stubs’ (*i.e.* as alanine). One turn of the 3XM spiral is shown in Fig. 6(*a* ▶).

The triple-mutant ODB has two regions of high flexibility and these are also the regions of the weakest electron density, indicating conformational flexibility. One region of high flexibility spans residues 213–218 (which includes the surface Cys216Ala mutation). Interestingly, this region (termed the B3 motif or loop) has been shown by mutagenesis to be important for the assembly of double hexamers in the context of full-length T-ag (Weisshart *et al.*, 1999 ▶). As this region was also difficult to trace in the wt T-ag OBD structure, we proposed previously that the inherent flexibility of this region may play a role in double-hexamer formation of full-length T-­ag (Meinke *et al.*, 2006 ▶). The other region of weak electron density occurs in the C-terminal portion, which includes the D256C mutation. A view of the electron density highlighting the ‘spiral’ interface which includes the F151C, D256C disulfide bridge is shown in Fig. 6 ▶(*b*). The C-terminal portion of T-­ag OBD has been shown to adopt alternate conformations in different structures of T-ag OBD (PDB entries 1tbd, 2fuf, 2ntc, 2if9, 2ipr, 2itj, 2itl and 2nl8; Meinke *et al.*, 2006 ▶, 2007 ▶; Luo *et al.*, 1996 ▶; Bochkareva *et al.*, 2006 ▶), indicating that it is also a region of high flexibility. However, it is clear that there is some structural rearrangement of the C-­terminal region in this structure relative to the wt structure, starting at amino acid 250. Indeed, the residues in this region are difficult to trace as a result of conformational flexibility. Nonetheless, the electron density for the disulfide bond connecting the protomers is clear.

Phe151 made extensive intermolecular contacts in the wt structure, so it is not surprising that some rearrangements occur when the large hydrophobic side chain is replaced by a smaller group. As a consequence, the wt structure buries larger surface areas (∼570 and 670 Å^2^, respectively) than the 3XM structure (∼480 and 570 Å^2^, respectively) as calculated using *PDBSUM* (Laskowski, 2009 ▶). Despite the indicated changes, the backbones of the two structures are virtually identical. Superposition of the two structures yields a root-mean-square deviation (r.m.s.d.) of 0.79 Å over 123 C^α^ atoms. Examination of the 3XM protein–protein interface shows that some contacts are absent in the mutant protein interface that were present in the wt structure. For example, a salt bridge between Lys214 and Glu177 that was observed in the wt structure is absent from the mutant structure. The structural rearrangements seen in our mutant spiral structure do not occur in the DNA-binding residues, which are primarily residues 147–155 and 203–207 (Simmons, Loeber *et al.*, 1990 ▶). Indeed, the r.m.s.d. between the wt and mutant structures is 0.139 Å (111 C^α^ atoms, residues 131–212 and 220–248). Therefore, in terms of DNA binding, our mutant spiral structure should behave similarly to the wt protein in its presumptively natural spiral form. Finally, although the triple mutant has reorganized a portion of the protein–protein interface, the disulfide cross-link, together with the other protein–protein interactions, are sufficient for formation of the crystallographic hexamer.

The crystal structure reported here demonstrates that the engineered cysteines in the T-ag OBD can form intermolecular disulfide bonds in a spiral hexamer. Furthermore, this mutant allows us to form higher order oligomers of T-ag OBD in solution, something that was not previously possible.

In addition to the crystallographic spiral observed for the SV40 T-ag OBD (Meinke *et al.*, 2006 ▶), spiral protein assemblies have been reported for other proteins involved in nucleic acid remodelling. Examples include the bacterial replication initiator DnaA (Erzberger *et al.*, 2006 ▶) and the eukaryotic ORC initiator from *Drosophila melanogaster* (Clarey *et al.*, 2006 ▶; reviewed by O’Donnell & Jeruzalmi, 2006 ▶). Protein assemblies known to form both flat rings and helical filaments include bacterial RecA (Yu & Egelman, 1997 ▶), archaeal RadA (Yang *et al.*, 2001 ▶) and *Methanothermobacter thermautotrophicus* MCM (Chen *et al.*, 2005 ▶). However, the ability to form a helical *versus* flat ring assembly for some systems appears to be a function of the protein-fragment length, as for the T7 Gp4 bacteriophage helicase domain; *i.e.* shorter fragments result in helical filaments (Sawaya *et al.*, 1999 ▶) and longer fragments in flat-ring structures (Singleton *et al.*, 2000 ▶; Toth *et al.*, 2003 ▶).

### 3.6. Conclusions

We now have a tool to probe the importance of oligomerization of T-ag OBD in a variety of biophysical studies by comparing the monomeric form of wt T-ag OBD with the higher order oligomer form of the disulfide-linked mutant. In addition, this represents a new reagent for studies of the oligomeric requirement for binding of cellular DNA replication factors to the T-ag OBD.

## Supplementary Material

PDB reference: SV40 large T antigen DNA-binding domain, 3qn2

## Figures and Tables

**Table 1:** Oligonucleotides for QuikChange mutagenesis

T-ag mutation	Oligonucleotides for QuikChange mutagenesis (their complements are not shown)
C216A	5′-CTGCTATTAATAACTATGCTCAAAAATTGGCTACCTTTAGCTTTTTAATTTGTAAAGGGG-3′
F151C	5′-AGTTTTTTGAGTCATGCTGTGTGTAGTAATAGAACTCTTGCTTGC-3′
D256C	5′-CCAGGTGGGTTAAAGGAGCATTGTTTTAATCCAGAAGAAGCAGA-3′

**Table 2:** Crystallographic data and refinement statistics for T-ag OBD_F151C, C216A, D256C_ Values in parentheses are for the highest resolution shell.

PDB code	3qn2
Beamline	NSLS X29
Wavelength (Å)	1.075
Space group	*P*6_5_
Unit-cell parameters (Å, °)	*a* = *b* = 83.933, *c* = 35.872, α = β = 90.000, γ = 120.000
Resolution (Å)	50–1.66 (1.72–1.66)
*R*_merge_ (%)	9.2 (50.0)
〈*I*/σ(*I*)〉	44.8 (7.1)
Completeness (%)	99.9 (99.5)
Multiplicity	21.6 (20.2)
Refinement	
Resolution (Å)	50–1.66
No. of reflections	17264
*R*_work_/*R*_free_	16.96/19.12
R.m.s. deviations	
Bond lengths (Å)	0.005
Bond angles (°)	0.920
